# Optimization of the basal medium for improving production and secretion of taxanes from suspension cell culture of *Taxus baccata* L

**DOI:** 10.1186/2008-2231-20-54

**Published:** 2012-10-15

**Authors:** Abolghasem Abbasi Kajani, Sharareh Moghim, Mohammad Reza Mofid

**Affiliations:** 1 Department of Biotechnology, Faculty of Advanced Sciences and Technologies, University of Isfahan, Isfahan, Iran; 2 Department of Bacteriology and Virology, Faculty of Medicine, Isfahan University of Medical sciences, Isfahan, Iran; 3 Department of Biochemistry, School of Pharmacy and Bioinformatics Research Center, Isfahan University of Medical Sciences, Isfahan, Iran

**Keywords:** *Taxus baccata*, Cell culture, Basal medium, Taxane production and secretion

## Abstract

**Background and purpose of the study:**

Taxol is one of the most effective anticancer drugs that isolated from *Taxus* sp. due to the slow growth of *Taxus* trees and low concentration of Taxol in the tissues, the biotechnological approaches especially plant cell culture have been considered to produce Taxol in commercial scale.

**Methods:**

We investigated the effects of basal medium type used in culture media on production of Taxol and other taxane compounds from cell suspension culture of *T. baccata* L. Briefly, five commonly basal media including Gamborg, Murashige and Skoog, Woody Plant, Schenk and Hildebrandt, and Driver and Kuniyuki medium were used for preparing separate suspension culture media. The intra- and extra-cellular yields of taxanes were analyzed by using HPLC after 21 days period of culturing.

**Results:**

The yields of taxanes were significantly different for the cultures prepared by different basal media. Moreover, the effects of basal medium on the yield of products differed for varius taxane compounds. Maximum yields of Baccatin III (10.03 mgl^-1^) and 10-deacetyl baccatin III (4.2 mgl^-1^) were achieved from the DKW basal media, but the yield of Taxol was maximum (16.58 mgl^-1^) in the WPM basal media. Furthermore, the secretion of taxanes from the cells into medium was also considerably affected by the type of basal medium. The maximum extra-cellular yield of Taxol (7.81 mgl^-1^), Baccatin III (5.0 mgl^-1^), and 10-deacetyl baccatin III (1.45 mgl^-1^) were also obtained by using DKW basal medium that were significantly higher than those obtained from other culture media.

## Introduction

**Figure 1:**
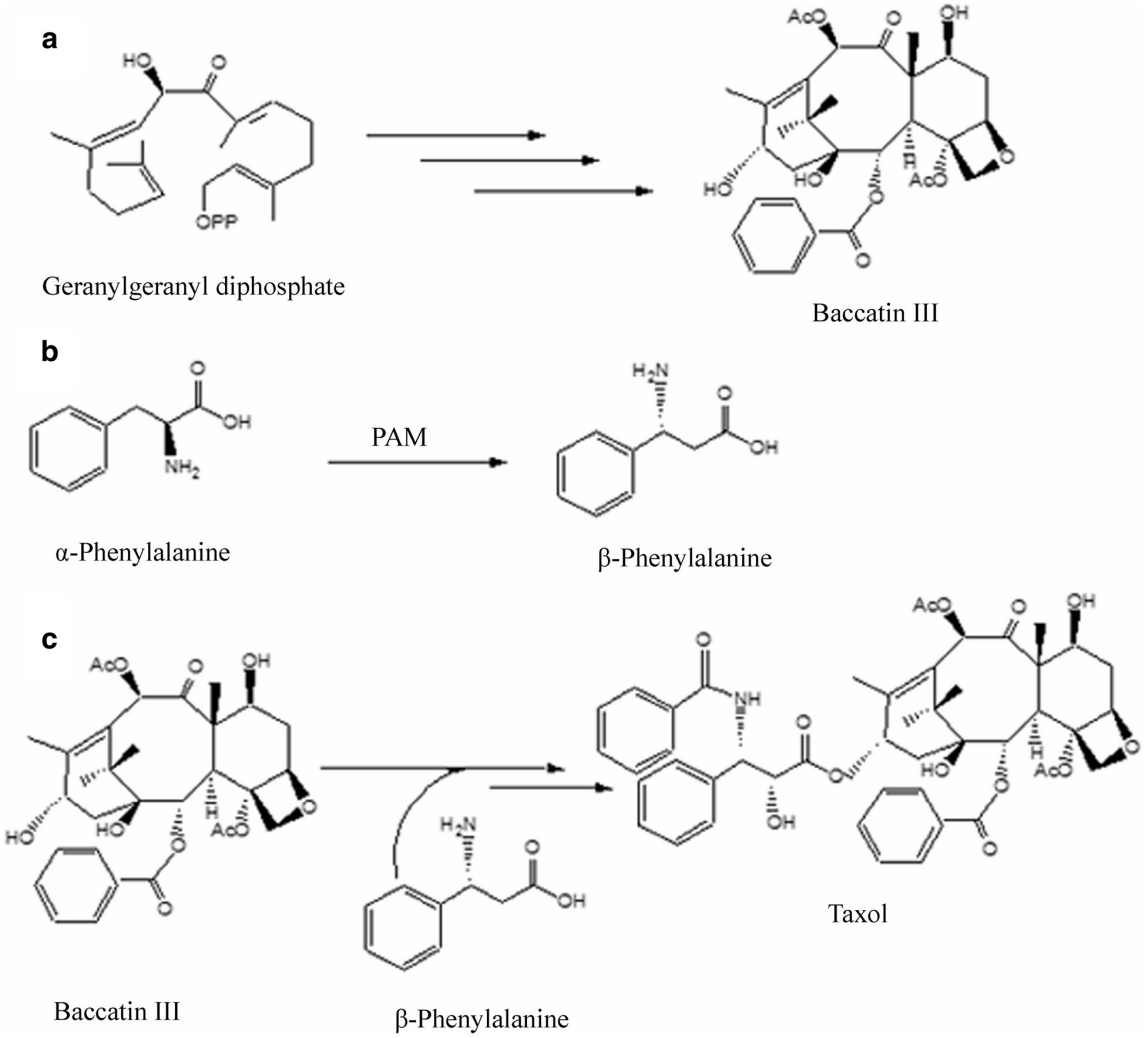
**Taxol biosynthesis pathway. a.**) biosynthesis of the taxane ring moiety from the terpenoid pathway. **b.**) conversion of α-phennylalaine to β-phennylalaine aminomutase (PAM) **c.**) attachement of the C-13 side chain to the Baccatin III and biosynthesis of Taxol.

*Taxus* species are known to produce a wide range of natural diterpenoids in approximately 350 identified forms named as taxoids [1]. Taxol is one of the best-known taxoids that was originally isolated from the bark of Pacific yew, *T. brevifolia*, in 1967 [2]. The Taxol biosynthesis pathway in yew contains approximately 20 distinct enzymatic steps (Figure 1). Due to the powerful effects of Taxol against a range of cancers and the expansion of clinical trials and treatments, its usage as an anticancer drug has been increased considerably [3]. This compound has traditionally been obtained from bark cuttings of the trees, which threatens the environment. On the other hand, the production of Taxol from original plant sources was limited due to the low abundance and slow growth of *Taxus* trees and low concentration of Taxol in the trees [4,5]. Therefore, investigating the alternative and non-destructive methods especially biotechnological approaches to improve Taxol productivity has been increased recently. Nowadays, large-scale plant cell culture is considered as one of the most promising, stable and long-term methods to produce taxoids [6,7]. In the most previous studies, media optimizitions for improving biomass production in the cell cultures of various *Taxus* species have been extensively studied. In this context, various basal media including Gamborg (B5), Murashige and Skoog (MS), Woody Plant Medium (WPM), Driver and Kuniyuki (DKW), and Schenk and Hildebrandt (SH) have been used for the initiation and the maintenance of callus and cell suspension cultures of *Taxus*[7]. The effects of various parameters such as combination and concentration of plant growth regulators, applying elicitors, use of organic solvents, sucrose feeding, and in situ extraction on the yield of taxanes production were also studied and the significant improvements have been obtained [4,8,9]. However, no significant study on the optimizition of basal medium for improving taxane synthesis and secretion from cell suspension culture of *Taxus* sp. was reported in the literatures according to our data. Therefore, we conducted a worthwhile study to investigate different basal media for improving the yield and also the recovery of taxanes from cell suspension culture of *T. baccata* L. 

## Material and methods

### Materials

Plant growth regulators [NAA (α-naphthalene acetic acid), kinetin and 2,4-D (2,4- dichlorophenoxy acetic acid)], nutrients and other plant tissue culture materials were purchased from *Duchefa* (Biochemie B.V., Netherlands). Organic solvents for taxane extraction were purchased from Merck (Germany). Standard of Taxol was prepared from Calbiochem (San Diego, CA). Standards of the other taxanes were purchased from Sigma (Germany).

### Plant material, media and culture conditions

*T. baccata* L. leaves used in this study were prepared from the tree present in the flower garden of Isfahan, Isfahan, Iran. To stablish the cell culture of *Taxus*, the homogenous calli were prepared from the leaf explants as previously described [10]. The young leaf explants were surface sterilized with immersing first in 70% ethanol for 1 min and then in sodium hypochlorite (1.5%) for 20 min. To remove disinfection liquid, the samples were washed for three times with sterilized autoclaved water. For callus induction, the leaf explants were cut into the segments with a length of 0.5 cm and cultured on the B5 medium supplemented with 2 mgl^-1^ NAA, 0.2 mgl^-1^ 2,4-D, 0.2 mgl^-1^ Kinetin, 500 mgl^-1^ PVP (polyvinyl-pyrrolidine), 25 gl^-1^ sucrose and 8 gl^-1^ agar. The samples were kept for about 30 days at 25°C in darkness. To obtain a homogenous callus, several subcultures of calli (up to 10 subcultures) were prepared in the same medium. For establishing the cell suspension culture, five different liquid media were prepared by using different basal media including WPM, DKW, B5, MS, and SH, that supplemented with the same composition of 2 mgl^-1^ NAA, 0.2 mgl^-1^ 2,4-D, 0.2 mgl^-1^ Kinetin, 500 mgl^-1^ PVP, and 30 gl^-1^ sucrose. Finally, 2 g of fresh friable homogenous calli were added to the 50 ml of the each liquid medium in 250 ml flasks and incubated for 21 days in darkness at 25°C with shaking at 120 rpm.

### Evans blue staining

Cell membrane permeability was analyzed using Evans Blue staining as previously described by Qiu et al. [11]. For this aim, the cells present in 10 ml of suspension culture were separated from the medium at days 14 and 21 of the period by centrifugating at 100 g for 10 min and washed carefully with 50 mM phosphate buffer (pH 5.8). The cells were then dried in dry filter paper and transferred into a tube and stained with 0.15% (w/v) solution of Evans Blue for 5 min. To remove the excess dyes, the cells were refiltered and washed with phosphate-buffered saline (PBS). The stained cells (50 mg) were collected and resuspended in 5 ml solution consisting of 1.0% (w/v) Sodium dodecyl sulfate (SDS) and 50% (v/v) methanol for 30 min at 50°C. The solution was finally analyzed with respect to its absorbance at 600 nm after cooled down to room temperature.

### Taxane extraction from the medium

The extraction process was carried out as previously described [12]. The cells and medium in the culture flasks were separated by filtration. The cell-free media were extracted carefully with methylene chloride (1:1, v/v) by shaking for 2 h. The organic phase was separated from the aqueous phase and concentrated in a rotary (Heidolph, Germany). The residue was re-dissolved in 1 ml methanol and filtered through a 0.22 μm filter before being subjected to reverse-phase HPLC analysis.

### Taxane extraction from the cells

Powdered dried cells were extracted by ultrasonication in 5 ml methanol for 40 min. The homogenates were filtered and the extracts were evaporated at 50°C to dryness under vacuum with a rotary evaporator. Residues were re-dissolved in 1 ml methanol and filtered for subsequent HPLC analysis.

### Analysis of taxanes

The taxane content in the methanol solutions were analyzed by reverse phase HPLC (Sykam) with UV detection at a wave length of 227 nm. The HPLC column used in the analysis was Kromasil C18, 250 mm × 4.6 mm and 5 mm packing (Alltech, USA). The mobile phase consisted of isocratic (at constant concentration) methanol and water (70:30, v/v), and the flow rate was 1 mlmin^-1^. The injection volume was 20 μl. The quantification of taxane compounds was determined based on external standards of genuine 10-deacetyl baccatin III (10-DEBA), baccatin III and Taxol.

### Statistical analysis

Effects of different basal medium used in the cell culture on the production and release of taxane compounds (10-DEBA, Baccatin III and Taxol) from *T. baccata* cells were investigated based on the experiment in a completely randomized design. All treatments were performed in triplicate and the results were analyzed by using MSTATC and Excel software. Statistical differences were analyzed by Student’s t tests, and a P value of less than 0.05 was assumed to be statistically significant.

## Results and discussion

### Taxane production from different media

Several studies have been extensively investigated the optimum conditions for production of Taxol in cell suspension cultures of *Taxus* and different improvements have been reported [4,7]. More commonly studies include optimizing cultural conditions, screening cell lines with high growth rate and productivity, optimizing the cell growth and production media, induction of secondary metabolite pathways by elicitors, the use of two-phase culture systems, and the immobilization techniques [7]. Different components of the medium especially carbohydrate source, Phytohormones, and precursors of the pathway, were analyzed with respect to their combinations and concentrations in the medium to obtain efficient production media. Different basal media such as B5, MS, SH and WPM have been employed for initiation and maintenance of callus cultures [13]. However, there is no noticable report in the literatures about the relationships between the basal medium used in cell culture with synthesis and secretion of the taxane compounds from *Taxus* cells. It seems that the concentration and composition of the nutrients in the medium not only affect on the growth and viability of cells, but could also be altered the synthesis of metabolites. Therefore, we investigated the yields of taxanes production by using different basal media including B5, MS, SH, WPM and DKW in cell suspension culture of *T. baccata* L.

The results (Table 1) showed the significant differences in average yields of taxane compounds produced by different culture media. The maximum yield of taxanes (28.6 mgl^-1^) obtained from the DKW basal media. This value was 2.7 fold higher than that the case of using SH basal medium (10.61 mgl^-1^) which presented the lowest productivity among the studied media. These results confirmed the significant effects of the composition and concentration of nutrients in the medium on the production of taxane compounds.

**Table 1:** **The yields of taxane compounds from different suspension culture media of *T. baccata *L**

**Basal medium**	**Taxol**	**Baccatin III**	**10-DEBA (mgl**^**-1**^**)**	**Total**
	**(mgl**^**-1**^**)**	**(mgl**^**-1**^**)**		**(mgl**^**-1**^**)**
WPM	16.58	3.9	3.92	24.4
DKW	14.37	10.03	4.2	28.6
MS	8.66	3.24	2.8	14.7
SH	5.82	2.69	2.1	10.61
B5	6.42	2.5	3.28	12.2

WPM, Woody Plant Medium; DKW, Driver and Kuniyuki; MS, Murashige and Skoog; SH, Schenk and Hildebrandt; B5, Gamborg.

Altought the media containing DKW basal medium produced highest yields of Baccatin III (10.3 mgl^-1^) and 10-DEBA (4.2 mgl^-1^), the maximum yield of Taxol (16.58 mgl^-1^) obtained from the media containing WPM basal medium. The results indicated that the composition and concentration of nutrients not only affect the taxanes production, but also had different levels of effectiveness on the production of various taxane compounds. Based on the results, it seems that different media could be stablished for efficient production of various taxanes. In general, maximum yields of the desired taxane could be obtained by using either DKW or WPM basal medium. The DKW basal media specifically produced higher amounts of Baccatin III compared to the other culture media. Therefore, it seems that DKW basal medium is suitable to stablish efficient culture media in order to synthesis of Baccatin III and 10-DEBA required for semisynthesis of Taxol. DKW and WPM basal media have been recommended for cell culturing of woody plants. Therefore, the high yields of taxanes may be related to appropriate supplying the nutritional requirements of *Taxus* cells in these media. Moreover, optimizing the basal medium in combination with appropriate elicitors could be resulted in higher yields of the products.

### *Taxane secretion from different media*

The accumulation of Taxol in *Taxus* cells leads to feedback inhibition and degradation of the product [14]. Different studies indicated that in situ removal of Taxol from suspension cell culture of *Taxus* led to the improvement in Taxol productivity [10,12,15]. Moreover such an approach also decreases the accumulation of taxane compounds within the cells, which could be considered themselves as toxic for plant cells [16]. On the other hand, finding a way to increase the secretion of taxanes from the cells into culture medium has been considered as an attractive approach in large-scale continuous production systems of taxanes [10]. This could facilitate downstream processing of the product as well as decreasing the costs needed to lyse the cells.

The use of different mechanical and chemical stimuli such as ultrasound [17], lanthanum salt [18], and organic solvents [11,17] were extensively investigated in the previous studies to induce secretion of taxane compounds from the cells in suspension culture of *Taxus*. However, most of these approaches tend to cause significant growth depression via inhibitory effects on viability and growth of the cells. Therefore, investigating a way to increase secretion of the products from the cells without inhibitory effects on the growth and productivity of cells is very essential.

Our results (Table 2) showed that the amounts of taxane compounds which secreted from the cells significantly differ between the culture media. Maximum extracellular taxanes (14.26 mgl^-1^) was obtained by using DKW basal medium that was approximately 50% of the total yield of taxane production in this culture medium. The value was 2.73 fold of that obtained from MS basal medium (5.23 mgl^-1^) which presented the minimum extracellular yield. These results obviously indicated that the taxane secretion from the cells could be considerably affected by the composition and concentration of the nutrients in the culture medium.

**Table 2:** **Effect of using different basal medium in the suspension culture of *Taxus *on inducing extracellular taxane portion**

**Medium**	**Taxol**		**Baccatin III**		**10- DEBA**		**Total**	
	**mgl**^**-1**^	**%**	**mgl**^**-1**^	**%**	**mgl**^**-1**^	**%**	**mgl**^**-1**^	**%**
WPM	7.68	46.32	1.38	35.38	1.33	33.93	10.39	42.58
DKW	7.81	54.35	5.0	49.85	1.45	34.52	14.26	49.86
MS	3.31	38.22	0.78	24.07	1.14	40.71	5.23	35.58
SH	3.59	61.68	1.15	42.75	0.86	40.95	5.6	52.78
B5	4.28	66.67	2.09	83.6	1.4	42.68	7.77	63.69

WPM, Woody Plant Medium; DKW, Driver and Kuniyuki; MS, Murashige and Skoog; SH, Schenk and Hildebrandt; B5, Gamborg.

Among the treatments, the culture media containing DKW basal medium also gave the best results with respect to the extracellular Taxol (7.81 mgl^-1^), this value was 54.35% of the total yield of Taxol in this culture medium. The maximum extracellular yields of Baccatin III (5.0 mgl^-1^) and 10-DEBA (1.45 mgl^-1^) were also achieved from the media containing DKW basal medium. Althogh maximum yield of Taxol production obtained by using WPM basal medium, only 46.32% of the product (7.68 mgl^-1^) was secreted into the medium. These results indicated that the optimum culture conditions for taxanes production may be different with the conditions for improved secretion of the products from the cells. Further studies on the composition of culture medium could be useful to achieve both higher yield and secretion of taxanes.

Although B5 basal media produced significantly lower yield of extracellular Taxol (4.28 mgl^-1^), these media showed maximum percent of Taxol secretion (67%). The secretion percents of Baccatin III and 10-DEBA were also higher in the culture media containing B5 basal medium. Ketchum et al. [19] previously studied the effects of basal salt mixtures on callus growth of three cell lines of *T. brevifolia* Nutt. They reported that Gamborg's B5 major salts provided significantly better growth than all other salt formulations tested (Anderson, Gresshoff and Doy (DBM2), MS, Nitsch and Nitsch (NN), Kao and Michayluk (KM), B5, and Litvay (LM). The results from present study indicated that the optimum condition for effective production of taxanes is different with the condition for optimal cell growth. It seems that a two-stage culture system where the cells are first specially cultured for biomass production and then transferred to a favorable medium to produce taxanes is an effective strategy for enhancing taxanes production.

### Cell membrane permeability in different media

Integrated cell membrane is impermeable for macromolecules such as Evans Blue [11]. Therefore, the effects of different treatments on the loss of cell membrane integrity could be assayed by monitoring the amount of the dye entering to the cells.

Evans Blue staining of *Taxus* cells separated from different suspension media during the culture period showed that the permeability of the cells had not been affected by the culture media significantly (data not shown). Thus, the improved secretion of the products from the cells occurred in some of the media could not be attributed to enhanced permeability of the cells. It seems that the improved secretion of the products from the cells may be related to the physiological events such as activating the transporter agents in cell membranes. In this context, further studies may be useful on the relationships of the medium composition with RNA or protein expression patterns and also with functions of the membraneous transporters.

## Conclusion

In conclusion, the present study suggested that using different basal media in suspension cuture of *Taxus* cells could be resulted in different yields of taxanes production. The results also indicated that the basal medium used in the suspention culture medium could be affected the secretion of the products from the cells into the medium. Moreover, different basal media affected the production of each taxane compound at different levels of effectiveness. Based on the results, it seems that using DKW basal medium in combination with appropriate plant hormones could be resulted in efficient production of taxane compounds. Finding the medium with efficient production and secretion of appropriate taxanes is accessible by further optimization. It seems that combining different elicitors with the optimum composition of nutrients and plant hormones could be resulted in synergic effects on the biosynthesis pathway of taxanes.

## Abbreviations

10-DEBA: 10-deacetyl baccatin III; HPLC: High-Performance Liquid Chromatography; 2,4-D: 2,4-dichlorophenoxy acetic acid; NAA: α- naphthalene acetic acid; PVP: polyvinyl pyrrolidine; B5: Gamborg; MS: Murashige and Skoog; WPM: Woody Plant Medium; SH: Schenk and Hildebrandt; DKW: Driver and Kuniyuki; PBS: phosphate-buffered saline.

## Authors´ contributions

AAK: preparing culture media and performing HPLC analysis. SM: Data analysis and preparing manuscript. MRM: Design of the project. All authors read and approved the final manuscript.
